# Acylcarnitines in Ophthalmology: Promising Emerging Biomarkers

**DOI:** 10.3390/ijms232416183

**Published:** 2022-12-19

**Authors:** Konstantinos Theodoridis, Helen Gika, Antigoni Kotali

**Affiliations:** 1 Laboratory of Organic Chemistry, School of Engineering, Aristotle University of Thessaloniki, 54124 Thessaloniki, Greece; 2 Laboratory of Forensic Medicine and Toxicology, Medical School, Aristotle University of Thessaloniki, 54124 Thessaloniki, Greece; 3 Biomic AUTh, Center for Interdisciplinary Research and Innovation (CIRI-AUTH), Balkan Center B1.4, 57001 Thessaloniki, Greece

**Keywords:** carnitines, acylcarnitines, L-carnitine, metabolites, metabolomics, biomarkers, ocular diseases, ophthalmology

## Abstract

Several common ocular diseases are leading causes of irreversible visual impairment. Over the last decade, various mainly untargeted metabolic studies have been performed to show that metabolic dysfunction plays an important role in the pathogenesis of ocular diseases. A number of metabolites in plasma/serum, aqueous or vitreous humor, or in tears have been found to differ between patients and controls; among them are L-carnitine and acylcarnitines, which are essential for mitochondrial fatty acid oxidation. The metabolic profile of carnitines regarding a variety of diseases has attracted researchers’ interest. In this review, we present and discuss recent advances that have been made in the identification of carnitines as potential metabolic biomarkers in common ocular diseases, such as age-related macular degeneration, diabetic retinopathy, retinopathy of prematurity, central retinal vein occlusion, primary open-angle glaucoma, rhegmatogenous retinal detachment, and dry eye syndrome.

## 1. Introduction

It is well known that vision impairment and blindness have a significant negative effect on the quality of everyday life, with these disabilities posing a significant financial burden and becoming a global public concern [1]. Recently, it has been reported by WHO that, globally, at least 2.2 billion people have a near or distant impairment [1], whereas two years earlier, the number was up to 1.3 billion [2]. The majority of them are over 50 years of age, although vision loss can affect people of all ages. Fortunately, drug discovery for eye diseases has significantly advanced in the past decade. A variety of valuable ocular therapeutics comprising small molecular weight drugs, fixed dose combination (FDC), gene therapy, ocular sealants, and antibody fragment inhibitors of vascular endothelial growth factor (anti-VEGF) have been developed in the field of ophthalmology [2]. However, despite this progress, there are problems that need to be addressed. Specifically, in early stages, some ocular diseases, for example, the majority of retinal diseases, have no clear symptoms, whereas during the progression of the disease, therapeutic options such as surgery, photocoagulation, or anti-VEGF agents have several limitations. Moreover, there are still pathophysiological mechanisms related to ocular diseases that have not been completely explained. In this context, there is a demand for a better understanding of ocular diseases and the development of novel biomarkers to enable earlier detection and diagnosis, treatment, progression, and prognosis.

Metabolomics focuses on the identification and quantification of key metabolites involved in metabolic perturbations related to certain pathophysiological states in a biological system, and it is a powerful tool for investigating the pathogenesis of a disease and the discovery of new biomarkers [3,4]. With the development of precision medicine, metabolomic analysis has been applied in ocular-derived matrices, and some interesting reviews have been recently published [5,6,7,8]. According to the literature, the metabolic profile of eye biospecimens under normal or several pathological conditions has been studied, and several metabolites have been identified to be involved in these. For example, taurine has been found to exhibit a protective effect against mitochondria-related metabolic impairments in the retinal pigment epithelium [9], whereas nicotinamide has shown to have neuroprotection on glaucoma [10]. Carnitines are a class of metabolites that have been recently shown to be related to ocular diseases [11,12,13,14,15,16,17,18,19,20,21,22,23,24,25,26,27]. Nevertheless, most clinical studies involving metabolomics studies are still preliminary and show important limitations.

Based on the above and on the fact that eyes are among the most metabolically active organs in the body and that several ocular diseases can be caused by metabolic dysfunction [28], and also given the important biological role of carnitines in numerous metabolic functions, we aimed to focus our study on the role of acylcarnitines in ocular diseases and investigate their potential as early diagnostic or prognostic biomarkers of pathological states leading to vision impairment. Thus, in this review, we present the progress that has been made regarding the application of carnitine derivatives as potential biomarkers in common ocular diseases. Herein the importance of the role of carnitines in human health are discussed and, thereafter, their connection with ocular diseases and the recent advances that have been made in the identification of carnitines as metabolites in common eye diseases. Finally, the conclusions and future directions are discussed.

## 2. Methodology

A PubMed/MEDLINE, Google Scholar and SciFinder literature search for English language articles was performed using the terms “retinal disease”, “age-related macular degeneration”, “diabetic retinopathy”, “retinopathy of prematurity”, “glaucoma” AND “metabolomics” OR “carnitine metabolite” OR “acylcarnitines” OR “metabolic profiling”. The abstracts were reviewed and, among them, the relevant articles were retrieved for comprehensive evaluation. References cited in selected articles were also reviewed to identify additional relevant reports.

## 3. Carnitines Biological Role

**Figure 1:**
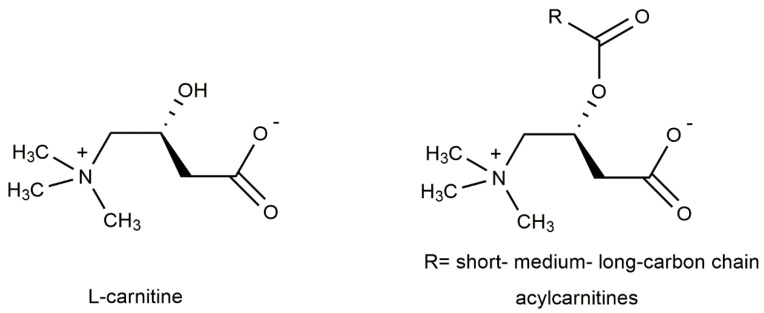
Chemical structures of L-carnitine and acylcarnitines.

**Figure 2:**
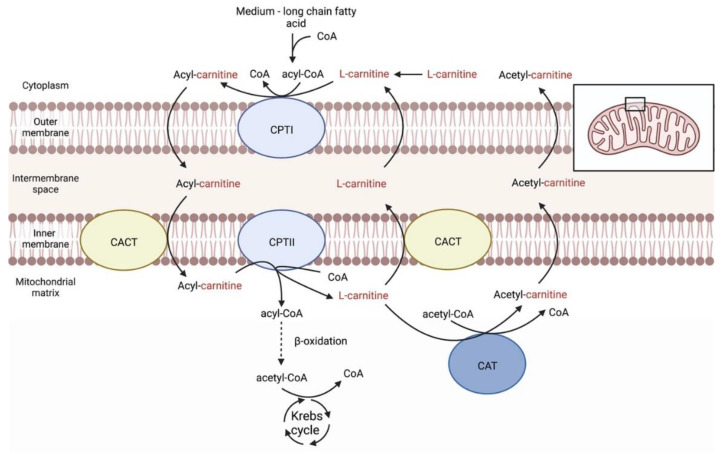
Carnitine shuttling system in eye tissues. L-carnitine seems to play an important role in the tissues of the eye, where cells of a muscular nature are present and may represent an important energy reserve after esterification. Specifically, medium- and long-chain fatty acids esterified to CoA are transesterified to acylcarnitine. This reaction is catalyzed by CPTI, an enzyme located in the outer mitochondrial membrane. It is possible for acylcarnitine to diffuse across the outer mitochondrial membrane and, then, to be transported through the inner membrane via CACT. CPTII, located in the inner mitochondrial membrane, catalyzes the formation of acyl-CoA from acylcarnitine in the mitochondrial matrix. Afterwards, acyl-CoA is catabolized in a process called β-oxidation and this results in the production of acetyl-CoA which enters the Krebs cycle. L-carnitine can be removed from the mitochondrial matrix via CACT. Alternatively, the enzyme CAT is responsible for the transfer of short-chain fatty acids from CoA to L-carnitine, and the newly formed acylcarnitine is exported into the cytosol by CACT. Therefore, L-carnitine is necessary for the transport and oxidation of medium- and long-chain fatty acids and the regulation of the availability of unbound CoA within the mitochondrial matrix, while, at the same time, it can be used as a reservoir for excess acetyl groups generated during fatty acid oxidation. CoA, coenzyme A; CACT, carnitine-acylcarnitine translocase; CAT, carnitine acetyltransferase; CPTI, carnitine palmitoyltransferase I; CPTII, carnitine palmitoyltransferase II.

L-carnitine is a quaternary ammonium compound known as L-3-hydroxy-4-aminobutyrobetaine or, alternatively, as L-3-hydroxy-4-*N-*trimethylaminobutanoic acid (Figure 1). It is also known as levocarnitine and vitamin BT. L-carnitine plays important role in physiological reactions throughout the body, including sugar aerobic metabolism and oxidative phosphorylation. In addition, L-carnitine has antiapoptotic, antioxidative, and osmoregulatory properties, which may be useful in the treatment of ocular pathologies. Being an essential metabolite, carnitine is absorbed from the diet, but it is also synthesized in the kidney, liver, and brain. It transports fatty acids across the mitochondrial membrane to undergo β-oxidation and produce energy (Figure 2). In animal tissues, L-carnitine concentrations are relatively high, typically between 0.2 and 6 mmol/kg, with almost being all in the heart and skeletal muscle [29]. While carnitine levels in the human ocular tissue are unknown, animal studies indicate that carnitine is differentially distributed within the eye with the highest concentrations are reported in the iris, ciliary body, and the choroid-retina [30].

Carnitine acyl esters, known as acylcarnitines, are essential for the oxidative catabolism of fatty acids and, consequently, for maintaining energy homeostasis in the human body [30,31]. Acylcarnitines are characterized as short, medium, and long chain depending on the size of the acyl group [32,33]. Very recently, a useful and extensive review of acylcarnitines has been published by Damprova et al. [33]. The authors provide a detailed description of acylcarnitines’ identity, nomenclature, classification, biochemistry, pathophysiology, supplementary use, potential drug targets, and clinical trials. They also summarized these updates in the Human Metabolome Database, which now includes information on the structures, chemical formulae, chemical/spectral properties, descriptions, and pathways for 1240 acylcarnitines.

Carnitine and its derivatives have been found to be related to a great variety of diseases. For example, recently they have been studied as a therapy or protective agent for many neurological diseases and neurotoxicity [34], while it has also been shown that some analogues are related to cardiovascular events [35]. Furthermore, a decreased level of hydroxydecanoylcarnitine and methylglutarylcarnitine has been suggested to be associated with the risk of metabolic syndrome [36], whereas lauroylcarnitine has been implicated as a mediator of obesity-induced inflammation [37]. Moreover, the circulating levels of bile acids and carnitine are differentially altered in patients with primary biliary cirrhosis [38]. Recently, the US Food and Drug Administration approved L-carnitine, along with short-chain acylcarnitines (acetylcarnitine and propionylcarnitine as a dietary supplement) [33].

## 4. Carnitines in Ocular Diseases

Over the last decade, several metabolomic studies regarding ocular health and disease have appeared in the literature. These studies have highlighted several metabolite species playing a role in the onset or progression of such conditions. In these cases, either targeted [13,17,18,19,21,24,25,27] or untargeted metabolic profiling methodologies [11,12,14,15,16,20,26] have been followed to study blood samples and, in fewer cases, ocular-derived specimens, such as aqueous humor, vitreous humor, and tears.

**Figure 3:**
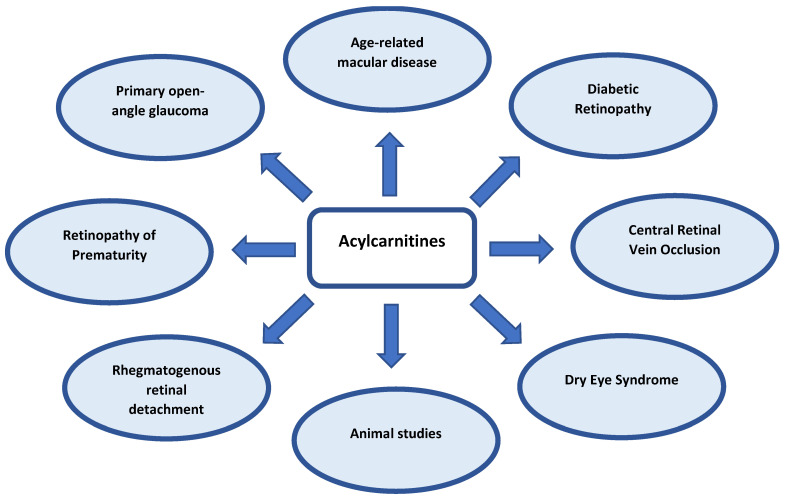
Acylcarnitines as biomarkers in common eye diseases.

Based on an untargeted approach, several classes of molecules have been identified as important to these conditions, such as amino acids, carbohydrates, phospholipids, and nucleotides, but also L-carnitine and various short-, medium- and long-chain acylcarnitines. In the cases where targeted metabolic profiling is followed, a set of preselected acylcarnitine species are measured in the samples. The preferable analytical technique is mass spectroscopy (MS) or tandem mass spectrometry (MS/MS), which, in most cases, are applied in hyphenation to liquid chromatography (LC-MS or LC-MS/MS) [12,15,16,18,20]. It is well known that MS identifies metabolite species on the basis of their mass/charge ratio (*m/z*), whereas MS/MS allows for the use of two or more stages of mass analysis to focus on the fragmentation of an ion within a mixture in order to enhance resolution and accuracy. Separation of the analyte via LC is used to facilitate the identification and quantification of the analyte. There are, however, cases where direct infusion to MS is applied (FIA-MS, or FIA-MS/MS) [13,17,22] without prior chromatographic separation. Due to the fact that carnitines contain a hydrophobic chain and a charged moiety, both reverse phase (RP) and hydrophilic interaction liquid chromatography (HILIC) have been used [26]. Nevertheless, the findings suggest that carnitine and its derivatives are highly implicated in common ocular dysfunctions. Table 1 summarizes the studies that imply untargeted or targeted metabolic profiling and that have indicated carnitine and/or its derivatives as biomarker of ocular diseases, while these are discussed in the following sections. A comprehensive scheme illustrating all the ocular diseases that have been reported to be related to carnitine metabolism is given in Figure 3.

### 4.1. Age-Related Macular Degeneration (AMD)

AMD is a disease that affects central vision progressively and is the major cause of visual impairment in people over 60 years of age. It constitutes 8.7% of worldwide total blindness, affecting approximately 150 million globally, and this number is projected to incline to 288 million by 2040 [39]. AMD is classified mainly as early stage (small-medium drusen deposits), intermediate (larger drusen deposits and/or retinal pigmentary changes), and late AMD. The latter is subdivided into two categories: (i) geographic atrophy or dry AMD, characterized by a gradual degeneration of retinal cells, and (ii) neovascular or exudative or wet AMD, in which new choroidal blood vessels are formed and proliferate, leading to exudation, retinal edema, hemorrhages, and central vision loss [40]. A combination of risk factors, including age, genetics, and environmental factors, such as tobacco use, a low dietary consumption of antioxidants, and hypercholesterolemia, leads to AMD [41]. Although several factors associated with the development of AMD have been identified, the etiology and the pathogenesis of the disease have not been fully delineated. Moreover, the interactions among cells and the exchange of metabolites between the retina, the retina pigment epithelium, and the choroid complex highlight the significance of finding metabolic local and systemic biomarkers which are indicative of the disease and improve the outcome. Therefore, AMD progression is related with metabolic dysfunction, and alterations in the level of metabolites, such as carnitines, might act as a diagnostic biomarker for its development.

Recently, a metabolomic study conducted by Mitchell et al. compared metabolite levels in plasma samples obtained from patients with intermediate AMD (IAMD), neovascular AMD (NVAMD), and non-AMD controls [15]. The aforementioned levels of metabolites among all groups were compared. By using a liquid chromatography-mass spectrometry (LC-MS) platform to analyze samples, and partial least-squares discriminant analysis (PLS-DA) and linear regression (LR) to identify discriminatory metabolic features, six acylcarnitines were found to meet the significance criteria. Among them, two carnitines (specifically linoleyl and linolenyl carnitine) were nearly 1.5-fold (1.63 and 1.49, respectively) higher in AMD patients compared to disease-free controls. Four carnitines (heptadecanoyl carnitine, 11Z-octadecenylcarnitine, glutaconylcarnitine, and stearoylcarnitine) were between 1.7- and 2.10-fold (2.08, 1.74, 1.75, 1.83, respectively) higher in NVAMD patients compared to IAMD patients. It should be mentioned that five of the acylcarnitines belong to medium- or long-chain acylcarnitine species. Furthermore, seven additional discriminatory features were identified with a medium or high confidence match with acylcarnitines, which presented higher levels along with more advance disease. Regarding metabolic pathway analysis, the carnitine shuttle pathway was the one that was significantly altered in all comparisons (AMD vs. control, IAMD vs. control, NVAMD vs. control, and NVAMD vs. IAMD). This study reveals the fact that plasma levels of certain acylcarnitines were not only higher in AMD patients and NVAMD patients compared with controls, but also in NVAMD patients compared with IAMD patients [15].

Furthermore, Mitchel et al. previously reported a metabolomic analysis of plasma samples obtained from NVAMD patients and healthy controls. Among the plethora of metabolic features analyzed, 39 of them were annotated with confidence, from which multiple were carnitine species. Using LC-MS/MS analysis, the identity of five acylcarnitine intermediates was confirmed. The plasma levels of the aforementioned five long-chain acylcarnitines (9-hexadecenoylcarnitine, heptadecanoylcarnitine, 11Z-octadecenylcarnitine, L-palmitoylcarnitine, and stearoylcarnitine) were significantly higher, approximately 2-fold (2.17, 2.16, 2.03, 1.95, and 1.65, respectively), in NVAMD patients compared to healthy controls. Furthermore, metabolite pathway analysis showed that the carnitine shuttle pathway was significantly changed in NVAMD patients [12].

Two more case–control studies have addressed altered plasma acylcarnitines in AMD patients. The first study, conducted by Luo et al., was an untargeted metabolomic study. It compared metabolomic features in the morning plasma of Chinese patients with wet AMD and healthy controls. By using ultra-high-pressure liquid chromatography and quadrupole-time-of-flight mass spectrometry (UHPLC-Q-TOF MS) to detect metabolic differences and multidimensional statistical methods to analyze samples, 10 metabolites were identified to differ significantly between the two groups of patients. Among them, L-palmitoylcarnitine was found to be lower in the group of patients with wet AMD (fold change 0.79) (variable importance for projection (VIP) >1, *p* < 0.05) [26]. The second case–control study by Chao de la Barca et al., which was based on a targeted metabolomics approach, compared plasma samples from patients with exudative/wet AMD and healthy controls. Among the 188 metabolites that were analyzed, only six remained statistically significantly altered after Benjamini–Hochberg correction, and two of them were carnitines, specifically free L-carnitine (C0) and valerylcarnitine (C5). The concentration of both carnitines was statistically increased in the plasma of AMD patients compared to controls and, in fact, valerylcarnitine was approximately 1.4 more concentrated in the blood of AMD individuals [13].

In another study conducted by Han et al., metabolomic changes in the aqueous humor of patients with wet AMD, in comparison to controls without AMD that underwent cataract surgery, were investigated. The untargeted metabolomics study, performed with the use of UHPLC-MS/MS and univariate analysis, showed 18 statistically significantly altered metabolites. Among them, two carnitines were identified, namely free L-carnitine (C0), which was decreased in the wet AMD group (approximately 0.72-fold change), and deoxycarnitine, which was increased in the patient AMD group (almost 1.87-fold change) compared to the control group. In addition, since the aforementioned molecule is the precursor substrate for carnitine biosynthesis, it should be mentioned that 6-N-trimethyl-L-lysine (TML) was significantly higher in the aqueous humor of AMD patients (1.84-fold change) compared with non-AMD controls. Therefore, alterations in free L-carnitine, deoxycarnitine, and TML suggest a disturbance in the carnitine pathway associated with mitochondrial dysfunction and fatty acid metabolism, possibly contributing to the pathogenesis of AMD [14].

### 4.2. Diabetic Retinopathy (DR)

DR, being a common complication of diabetes mellitus (DM), constitutes the main cause of blindness in the adult, working-age population [42]. It affects approximately 103 million patients worldwide, and the number is estimated to rise to 160.5 million patients in 2045 [43]. The disease leads to visual impairment, having a devastating impact on patients’ quality of life [44]. DR is categorized mainly into two stages: non-proliferative DR (NPDR), characterized by microaneurysms, haemorrhages, cotton-wool spots, and intra-retinal microvascular anomalies (IRMAs); and proliferative DR (PDR), which is characterized by neovascularization and/or vitreous haemorrhage [45]. The risk factors for DR include poor glycemic control and, thus, hyperglycemia, as well as hypertension and hyperlipidemia [46]. Therapeutic strategies, such as anti-VEGF injections, are limited and the understanding of the DR pathogenesis is still incomplete. Therefore, further investigation should be undertaken and new metabolites, such as carnitines, could play an important role in the early detection of the disease and the optimization of patient care. 

In a recent study conducted by Sumarriva et al., metabolic profile differences of plasma samples between patients with type 2 DM, with DR and without DR (diabetic controls), and between patients with PDR and NPDR have been shown. LC-MS analysis was performed; features were selected using PLS-DA (VIP ≥ 1.5) and were significantly associated (*p* < 0.05) in a Wilcoxon rank sum test. Several metabolic features were found to differ significantly between DR patients and diabetic controls, four of which constituted key contributors to the pathways analysis differences detected in the study; dehydroxycarnitine was among them and its concentration was found to be significantly increased in DR patients (*p* = 0.0069). Regarding PDR, pathway analysis revealed alterations in the β-oxidation of saturated fatty acids (*p* = 0.032), as well as fatty acid metabolism (0.038). Carnitine, being a key molecule in the metabolism and oxidation of fatty acids in mitochondria, was found to be statistically significantly increased in PDR patients in comparison with NPDR patients [14]. 

Furthermore, Yun et al. performed a metabolomics analysis in serum samples obtained from patients with type 2 DM. Specifically, patients were divided into three groups, i.e., NDR, NPDR, and PDR. Metabolites were quantified with the use of LC-MS and FIA-MS, and statistical analysis was performed with R software. The concentrations of several carnitines were found to be significantly different among groups and they could act as potential disease markers. In fact, propionylcarnitine (C3) and butyrylcarnitine (C4) showed significantly higher concentrations in the DR patient group compared with the NDR group (a 1.16- and 1.31-fold change, respectively), while the concentrations of dodecanoylcarnitine (C12), tetradecenoylcarnitine (C14:1), tetradecadienylcarnitine (C14:2), hexadecanoylcarnitine (C16), octadecenoylcarnitine (C18:1), and octadecadienylcarnitine (C18:2) were lower in the DR group (0.89-, 0.87-, 0.87-, 0.86-, 0.87-, and 0.87-fold change, respectively). Additionally, comparing serum samples from NPDR and NDR patients, the concentrations of three acylcarnitines, namely L-carnitine (C0), tetradecenoylcarnitine (C14:1), and hexadecanoylcarnitine (C16), were found to be lower in the NPDR group (0.96-, 0.88-, and 0.87-fold change, respectively). Comparing the concentrations of metabolites between PDR and NDR patients, three short-chain acylcarnitines (propionylcarnitine (C3), butyrylcarnitine (C4), and valerycarnitine (C5)) showed statistically higher concentrations in PDR patients (1.25-, 1.63-, and 1.23-fold change, respectively), while five long-chain acylcarnitines, tetradecenoylcarnitine (C14:1), hexadecanoylcarnitine (C16), octadecanoylcarnitine (C18), octadecenoylcarnitine (C18:1), and octadecadienylcarnitine (C18:2), showed lower concentrations in the PDR group (0.84-, 0.82-, 0.83-, 0.81-, and 0.81-fold change, respectively). Moreover, among the metabolites that statistically differed between PDR and NDPR patients, the medium-chain pimelycarnitine (C7:DC) was found to be elevated in the PDR group, and its concentration was reported to be 1.10-fold higher. Finally, the concentrations of sixteen metabolites showed differences when comparing both PDR and NPDR patients to NDR patients. Two acylcarnitines, namely tetradecenoylcarnitine (C14:1) and hexadecanoylcarnitine (C16), were included among them, and their concentrations were lower in the PDR and NPDR groups than in the NDR group (0.87- and 0.86-fold, respectively) [15].

Interestingly, in a prior study by Paris et al., the content of metabolites in vitreous humor samples from patients with PDR and non-diabetic controls were compared. Initial analysis was performed in a first set of patients, and afterwards, the findings were validated through the analysis of a second set of patient samples. The concentrations of two medium-chain carnitines, specifically decanoylcarnitine (C10) and octanoylcarnitine (C8), were increased in the group of PDR patients compared to non-diabetic controls in both sets of patients (C10 with 1.7- and 1.4-fold changes and C8 with 2.2- and 1.7-fold changes in the first and second set of patients, respectively) [18].

### 4.3. Retinopathy of Prematurity (ROP)

ROP is a disease characterized by immature retinal vascular development and the presence of retinal ischemia and neovascularization, which can lead to retinal detachment and vision loss. Both prematurity and low birth weight are factors strongly related to a high risk of the disease [47]. It should be mentioned that ROP affects almost 100,000 children globally, according to an epidemiological study [47,48,49,50]. Indeed, the incidence and the severity of ROP is still high, and the pathogenesis is not yet fully understood. Therefore, metabolites, such as carnitines, could act as potential biomarkers and contribute to the early diagnosis and better understanding of the underlying mechanisms of the disease.

Recently, Yang et al. conducted a study based on a targeted metabolomics analysis of blood from premature infants, comprising two groups of cases: ROP patients and non-ROP controls [19]. UPLC-MS targeted analysis was applied for the measurement of malonylcarnitine. Subsequently, standard multivariate and univariate analysis was performed for interpretation of the results. Malonyl carnitine (C3DC) was found and confirmed to be statistically significantly different (*p* < 0.001) between ROP and non-ROP infants. C3DC constituted an independent strong risk factor for ROP, since the higher the value of the specific acylcarnitine, the higher the risk of ROP. Although a probable association is revealed between C3DC and the risk of ROP, this is not the case for the severity of the disease. In addition, the predictive ability of C3DC for ROP diagnosis was the best among the other discriminant metabolites (area under the curve (AUC) = 0.914, sensitivity = 97.5%, and specificity = 68.3%), showing that C3DC could be a potential biomarker for the diagnosis of ROP [19].

### 4.4. Central Retinal Vein Occlusion (CRVO)

CRVO is a common disease of the retinal vasculature which may lead to visual loss, usually unilateral [51,52]. It is estimated to affect approximately 0.1–0.5% of the population. Its pathogenesis is associated with atherosclerosis of the central retinal artery, which is situated adjacent to the vein within the same adventitia, compressing and inducing stasis and thrombosis in the vein lumen, and leading to CRVO [52]. In this condition, there is an increase in intravenous hydrostatic pressure and a nonperfusion of the retinal capillaries, leading to a decrease in oxygen supply. The aforementioned alterations in the retinal blood flow result in ischemia and hypoxia-induced metabolic dysregulation [26,53]. Therefore, changes in the concentrations of metabolic substances and dysfunction of several pathways, among which is the carnitine shuttle pathway, could reveal the pathological mechanisms and the progression of the disease. 

A study conducted by Wei et al. performed a metabolomic analysis of the aqueous humor obtained from fifteen patients with CRVO and twenty patients who underwent cataract surgery (controls). UHPLC-MS/MS was used to identify the involved metabolites [26]. Butyrylcarnitine (*p* = 0.003) and deoxycarnitine (*p* value not reported) were found to be elevated and among the statistically significant metabolites in CRVO group. Furthermore, 6-N-trimethyllysine (TML), being the compound from which the biosynthesis of carnitines starts, was significantly altered in CRVO patients as well. A speculation that was suggested is that the dysregulated carnitine metabolism pathway might be involved in mitochondrial dysfunction in CRVO patients; however, regarding the pathogenesis of CRVO, any exact involvement of such metabolomic pathways is still unknown.

### 4.5. Primary Open-Angle Glaucoma (POAG)

POAG represents the most common form of glaucoma and it is a group of chronic optic degenerative neuropathies characterized by progressive impairment of retinal ganglion cells and visual field loss without an identifiable cause [54,55]. The risk of developing POAG is related to many factors, among which the most prominent one is elevated intraocular pressure (IOP) [24,55]. POAG affects approximately 57.5 million people and is the leading cause of irreversible blindness globally; it is projected to reach 111.8 million cases in 2040 [56]. Due to the slow and asymptomatic progression of the disease in the early stages, the diagnosis of POAG often does not occur until later when severe field damage and loss of central fixation are established [24]. Therefore, the asymptomatic nature of POAG, combined with the worldwide prevalence of the disease and its estimated dramatic increase in the ageing population in the following years, necessitate the need of discovering new strategies and possible systemic or topical biomarkers, such as carnitines, for an early stage accurate diagnosis.

Two studies compared the metabolic profiles of plasma taken from patients with POAG and from controls. In the first study conducted by Burgess et al., LC-MS was used for metabolic profiling analysis of plasma samples, and further statistical analysis of the data revealed several differentially expressed metabolites associated with POAG. Among the aforementioned metabolites palmitoylcarnitine (C16), a long-chain acylcarnitine, was found to be significantly elevated in the plasma of POAG patients in comparison with controls (*p* = 3.69^−10^) [20]. It is well known that palmitoylcarnitine is part of the carnitine shuttle pathway, functioning for the transport of fatty acids for β-oxidation in the mitochondria. The aforementioned findings are in accordance with an earlier study regarding the deficiency of carnitine palmitoyl transferase II and its connection with normal tension glaucoma [57]. However, since various factors could be related to the palmitoylcarnitine metabolism in the mitochondria, the elevated levels of palmitoylcarnitine in the plasma of POAG patients might be part of a more generalized defect of mitochondrial function and not specifically associated with POAG [20,58].

Similarly, in the second study, Leruez et al. compared the metabolic profiles of plasma samples obtained from patients with POAG and controls with cataract. FIA-MS was used for the analysis of metabolites, and statistical analysis revealed 18 significantly altered metabolites between the two groups, six of which were identified to be carnitines. More specifically, univariate statistical analysis (with the use of the Wilcoxon test) showed an increase in propionylcarnitine (C3) (fold change = 1.30, *p* = 0.0036) in the POAG group in comparison to controls, while octadecenoylcarnitine (C18:1) was decreased (fold change = 0.76, *p =* 0.0017) in POAG patients. In addition, multivariate analysis (with the use of the least absolute shrinkage and selection (LASSO) operator) showed an elevation in the concentration of butyrylcarnitine (C4), decanoylcarnitine (C10:1), and dodecanoylcarnitine (C12:1) in the group of POAG patients compared with cataract controls (fold change and *p* value are not reported for these metabolites). Both univariate and multivariate analysis identified octadecadienylcarnitine (C18:2) to be statistically significantly increased (fold change = 0.82, *p* = 0.00068) in the POAG group compared with controls [21]. The alterations in acylcarnitine concentrations delineate a perturbation in the fatty acid metabolism and are in accordance with the study of Burgess et al., where the long-chain palmitoylcarnitine was found to be increased. In addition, Rong et al. revealed five long-chain free fatty acids which were statistically significant in PACG patients but not acylcarnitines, and Mayordomo-Febrer et al. found elevated free fatty acid concentrations in the aqueous humor of their animal model (rats) of glaucoma [59,60]. Taking into consideration the significant role of carnitines in the transport of fatty acids in the mitochondria and the inherited disorders of fatty acid oxidation, it could be presumed that when the oxidation of fatty acids is incomplete, this results in a reflux from mitochondria into the blood stream of smaller acylcarnitines, which may be the cause of increased acylcarnitine levels [61]. Furthermore, the concentration of two long-chain acylcarnitines was found to be lower in POAG patients, which might be part of the ageing process, as it has been shown to decline with age in the blood of mice [62].

A recent study conducted by Buisset et al. compared the metabolomic profiles obtained from the aqueous humor of POAG patients with that of controls undergoing cataract surgery. Again, FIA-MS was used to identify metabolites, and multivariate and univariate statistical analysis was performed to discriminate metabolites between the two groups. Among the metabolites which were found to be discriminant using multivariate analysis, three short-chain acylcarnitines, namely acetylcarnitine (C2), propionylcarnitine (C3), buryrylcarnitine (C4), and free carnitine (C0), were included. The concentrations of the aforementioned carnitines were statistically higher in the aqueous humor of POAG patients compared to controls. Additionally, univariate analysis after correction for the false discovery rate (FDR) demonstrated that C2 and C3 were significantly higher than other metabolites. Metabolites, including carnitines, were ranked based on importance using the median value of VIP, as well as the adjusted p-value (C3: VIP = 1.56, *p* = 0.013; C2: VIP = 1.52, *p* = 0.029; C0: VIP = 1.32, *p* = 0.067; C4: VIP = 1.11, *p* = 0.087) [36]. The study by Buisset et al. was the first study to show elevated concentrations of short-chain carnitines (C2, C3, C4) and free carnitine in the aqueous humor of POAG patients. Furthermore, carnitine has shown neuroprotective properties, as well as antioxidant and antiapoptotic properties, in retinal cells in the eyes of mice with high intraocular pressure [63]. Therefore, an increase in the concentration of carnitine could be involved in the protection of retinal cells in the case of disease-related stress and production of reactive oxygen species. Additionally, the lack of long-chain acylcarnitines in the metabolic profile of the aqueous humor of POAG patients is not in favor of mitochondrial oxidation impairment in the cells obtained by the aforementioned fluid. On the contrary, a defect in the amino acid metabolism could be presumed, since the concentration of several amino acids, along with that of short chain acylcarnitines, which are associated with their degradation, is increased [37]. A previous study by Leruez et al. showed a higher concentration of C3 and C4 acylcarnitines in the plasma of POAG patients, demonstrating the important and systemic role of these short-chain acylcarnitines in the pathogenesis of POAG [21].

Additionally, Lillo et al. investigated the metabolomic composition of the aqueous humor of patients with POAG, comparing them with healthy controls [23]. This study confirmed the study of Buisset et al. regarding the changes in the levels of C0, C2, C3, and C4 that increased in glaucoma patients. In fact, C0, C2 and C3 were the most abundant carnitines, with concentration values of 9.8 μΜ, 1.7 μΜ, and 0.2 μΜ, respectively. However, the study of Lillo et al. showed an increase in the number of acylcarnitines whose concentration was above the detection limit, of which there were 13 in total; the levels of 10 acylcarnitines significantly increased in the group of glaucoma patients, whereas the concentrations of C4:1 and C14:2OH were similar between the two groups and the concentration of C10 decreased in the glaucoma samples. These alterations in the levels of acylcarnitines seem to be of mitochondrial origin, and the role of C10 carnitine, which was the one that decreased, should be evaluated further regarding its role in the functionality of the mitochondria. Indeed, in the past, the exogenous administration of this metabolite interestingly led to impairment in mitochondrial handling, as well as fatty acid oxidation and the inhibition of ketogenesis [23]. 

A recent study conducted by Rossi et al. enrolled POAG patients and healthy controls, and determined their levels of metabolites. Among them, acylcarnitines were identified with the use of direct infusion mass spectrometry (DI-MS). Following a multivariate statistical approach, the levels of tear acetylcarnitine (C2) were found to be statistically significantly lower (*p* = 0.008) in POAG patients compared with controls. Free carnitine (C0) showed a tendency towards lower levels (*p* = 0.05) in the POAG group of patients in comparison to the healthy controls [24]. Therefore, an easily accessible biofluid, such as tears, could be helpful in detecting molecules such as carnitines, and this correlation of POAG biomarkers and the progression of disease could lead to an early diagnosis and interesting aspect of investigation in future studies.

### 4.6. Rhegmatogenous Retinal Detachment (RRD)

RRD is a condition that is caused when fluid passes from the vitreous cavity through a tear of the retina into the subretinal space, specifically between the neurosensory retina and the underlying retinal pigment epithelium (RPE), leading to the separation of the two tissues. RRD is a significant cause of visual impairment and it will result in total blindness if the retina is not repaired properly [40]. Proliferative vitreoretinopathy (PVR) is a complication that might follow the RRD process and the most common reason for RRD repair failure. More particularly, PVR comprises the growth of cell membranes in the vitreous cavity and their contraction leads to retinal re-detachment and loss of vision, with poor visual outcomes even with repeated interventions [64]. 

A study conducted by Li et al. performed a metabolomic analysis of human vitreous samples obtained from patients with RRD, patients with recurrent retinal detachment and PVR, and controls (donor eyes) in order to identify novel potential biomarkers that will help to clarify the mechanisms and pathogenesis of the formation of RRD and PVR. Relevant metabolites were revealed via a LC-Q-TOF-MS method and multivariate statistical analysis, which was used to distinguish the metabolite concentrations that were significantly altered between patient groups. Specifically, only eleven metabolites were statistically different between eyes with RRD and PVR. Among them, carnitine was found to be decreased in both groups. In fact, the extent of the L-carnitine decrease was significantly more prominent in RRD samples than in PVR samples (*p* < 0.01 between the two groups) [25]. In addition, a dysregulation of pathways linked to inflammation was detected [5,25]. More particularly, in several reports, L-carnitine is associated with the control of inflammatory response, as well as the potential ability to suppress pro-inflammatory cytokines [65,66]. Therefore, an elevated level of L-carnitine could potentially inhibit inflammation. The study by Li et al. showed that the lower levels of L-carnitine may cause an amplification of inflammation development in both patient groups, RRD and PVR. Since the decrease in the concentration of L-carnitine was significantly more evident in RRD samples compared to those with PVR, this led to the fact that the inflammation is more serious in eyes with RRD alone [25]. Furthermore, this study showed that some of the identified metabolites were associated with proliferation of pathology-related cells, whereas others showed some anti-proliferation effects. It is stated that L-carnitine can suppress the proliferation and further differentiation of vascular smooth cells [67]; thus, it inhibits vessel growth and decreases the degree of visual loss. Interestingly, carnitine was elevated in PVR samples in comparison to those with RRD alone, which reveals a positive regulation made by the organism to inhibit the proliferation of pathological cells [25].

### 4.7. Dry Eye Syndrome (DES) 

DES is a multifactorial disease that affects the homeostasis of the tear film and, thus, the integrity of the ocular surface. In fact, tear film instability and hyperosmolarity can play an important etiological role in DES [68]. Carnitines seem to be involved in the regulation of tear film osmolarity since they are thought to have osmoregulatory properties protecting the affected cornea surface [27].

Pescosolido et al. conducted a case–control study using HPLC-MS, analyzing the presence of carnitine as well as its derivatives, i.e., L-acetylcarnitine and L-propionylcarnitine, in the tears of DES patients and healthy subjects. Comparing tear samples between the two groups, the concentrations of carnitine, L-acetylcarnitine, and L-propionylcarnitine were statistically significantly lower in the tears of DES patients (*p* < 0.05). Therefore, it has been suggested that tear film osmolarity might be regulated by carnitines which could have a protective effect preventing damage of the ocular surface. An imbalance in the concentration of carnitine molecules in the tear film is suggested to be connected with DES, and further studies should be conducted to address their protective role as osmo-protection molecules and their possible use as biomarkers of the disease early diagnosis [27,69]. 

### 4.8. Animal Studies

Metabolic perturbations and particularly alterations in the concentrations of several acylcarnitines have also been detected in animal studies related to ocular diseases. More specifically, in a study conducted by Yanshole et al., the metabolomic profiles of rat lenses were investigated by nuclear magnetic resonance (NMR) and HPLC-MS. The analysis regarding age-related changes in the metabolic composition of the lenses showed a decline in several metabolites, and among them, in carnitine. In fact, the most significant changes are observed between the ages of 1 and 3 months when the younger lenses of the Wistar rats are richer in metabolites compared to the older rats, since the period of enhanced metabolomic activity is terminated with the completion of the maturation process of the lens during the first month. A statistically significant difference was detected in the concentration of carnitine in the lens, which was decreased by approximately 100% between 1 and 3 months, and by 50% between 3 and 14 months. In addition, the concentrations of metabolites of Wistar rats were compared with those of senescence-accelerated OXYS rats. A statistically significant difference was found for carnitine in the OXYS lens, the concentration of which was on average 30% higher than in the Wistar lens. This could be explained by the fact that there is an excessive production of reactive oxygen species in tissues of the OXYS rats and, thus, the enhanced level of some metabolites, and among them of carnitine, which could be attributed to the compensatory response to the aforementioned oxidative stress [70].

Another animal study conducted by Kurihara et al. investigated genetically triggered hypoxia in retinal pigment epithelium (RPE) cells that could lead to photoreceptor atrophy in mouse models, thus trying to gain an insight into the pathogenesis of AMD. In fact, the aforementioned hypoxia-induced metabolic stress triggered alterations in the lipid metabolism of hypoxic RPE cells and, more particularly, in a group of medium- and long-chain acylcarnitines. Therefore, the identification of a group of acylcarnitines that were dysregulated from 3 to 14 days post induction could be informative for the early diagnosis and a better understanding of diseases, such as AMD, in which defects in RPE and photoreceptors are common [71].

Additionally, a study conducted by Rowan and coworkers investigated the contribution of dietary patterns that differ in the type of dietary carbohydrate on AMD in a mouse model. Given the fact that the consumption of a high-glycemic diet resulted in several AMD features, such as RPE hypopigmentation and atrophy and photoreceptor degeneration, whereas the consumption of low-glycemic dietary products did not, it is not unreasonable to think that switching from a high-glycemic diet to a low-glycemic diet could stop or reverse AMD features. The findings revealed an interaction between carbohydrate intake, AMD features, and the metabolome. In fact, among the metabolites which were statistically significantly different was C3-carnitine, which was found to be lower in the retina, plasma, or urine of affected mice, i.e., the ones that were given the high-glycemia diet. More specifically, C3-carnitine was a metabolite that performed nearly perfectly in ROC analysis regarding the ability of a metabolite to distinguish unaffected from affected retinas, and its plasma levels were statistically lower in affected individuals (AUC = 0.969, *p* = 0.00264). Therefore, C3-carnitine could act as one of several potential biomarkers for earlier diagnosis, prognosis, or evaluation of the efficacy of a new therapy, and provide a better understanding of the retinal maintenance and possible mechanisms of AMD features [72].

Finally, in the aforementioned study of Paris et al., metabolomic analyses were performed on ocular samples from mice with oxygen-induced-retinopathy (OIR) which exhibits several comparable pathological retinal features also observed in PDR. The metabolomic profile of the OIR mouse model was analyzed at the 17th day of age, i.e., the time of maximal pre-retinal neovascularization. Among the metabolites that were altered in OIR mouse eyes compared with the normoxic ones were three acylcarnitines, namely octanoylcarnitine (C8), propionylcarnitine (C3), and acetylcarnitine (C2), that were increased in OIR models by 3.0-, 86.4-, and 2-fold compared to controls. Thus, octanoylcarnitine was the metabolite which was altered in both human and mouse eye samples, while decanoylcarnitine concentration was below the limit of detection in the OIR mouse model [18].

## 5. Discussion

Metabolomics is an emerging and potentially powerful tool in ophthalmology research. Metabolic profiling studies that have been performed in the past ten years have demonstrated that among the various metabolites that were found to differ between patients with common ocular diseases and controls [6,7], there were also L-carnitine and various short-, medium- and long-chain acylcarnitines. Acylcarnitines have already been identified as important indicators in metabolic studies of many diseases, including metabolic disorders, cardiovascular diseases, diabetes, depression, neurologic disorders, and certain cancers, [34,35,36,37,38,73] and, interestingly, there is growing evidence on their role in ocular dysfunctions. 

According to the findings that have been presented above, the alterations in the levels of either free L-carnitine or certain acylcarnitines suggest a possible important role of the carnitine shuttle pathway in several ocular diseases. The aforementioned pathway is responsible for the transport of fatty acids, especially medium- and long-chain ones, into the mitochondria for the subsequent catabolism via β-oxidation, which is a process that requires acyl-CoA and leads to the esterification of L-carnitine to form acylcarnitine derivatives [12,74]. It is possible that changes in the levels of acylcarnitines and the impairment of the carnitine shuttle pathway indicate a subsequent dysfunction in the mitochondrial fatty acid metabolism [6,15]. Furthermore, there are links between fatty acid oxidation and the regulation of angiogenesis. It has been demonstrated that fatty acid oxidation is essential for maintaining redox homeostasis and preventing dysfunction in endothelial cells [75]. Moreover, the inhibition of fatty acid oxidation via carnitine palmitoyl transferase 1 (CPT1), a rate-limiting enzyme, repressed proliferation and neovascularization in endothelial cells and inhibited pathological ocular angiogenesis in mice [74]. NVAMD such as PDR are characterized by new vascular growth. Evidence showed that in both diseases, acylcarnitines are altered and these changes in fatty acid metabolism are associated with ischemia or neovascularization [16]. For example, it has been reported that long-chain acylcarnitine levels were increased in patients with neovascular AMD [12], whereas L-carnitine has the potential to protect the retina from ischemia-reperfusion injury [76]. Furthermore, it has been suggested that the perturbation of the carnitine shuttle pathway and the compromised mitochondrial function could decrease cellular capacity to handle reactive oxygen species, resulting in increased cellular dysfunction and cell death [12].

The eyes are organs that highly demand energy and mitochondrial fatty acid metabolism is expected to have an impact on their function. This impact has been investigated in retinal cells [27]. According to a retinal model of mitochondrial impairment, disorders in carnitine metabolism and in fatty acid β-oxidation may lead to retinopathy [77]. It is tempting to speculate that a possible decrease in free L-carnitine and an increase in various chain acylcarnitines may lead to compromised mitochondrial function. This could be explained due to the fact that the possible decreased level of L-carnitine found in studies may indicate the increase in esterification to generate acylcarnitine derivatives, while an incomplete oxidation of fatty acids results in a reflux from mitochondria toward the blood of various acyl-carnitines, especially smaller ones, as was the case in POAG patients [21,22,23], which may be the origin of increased acyl-carnitine levels. In fact, this phenomenon has been reported in inherited disorders of fatty acid oxidation [61]. On the other hand, long-chain acylcarnitines were also found downregulated in some studies in POAG or PDR patients, which is possibly in accordance with a senescence-like phenotype since the concentration of long-chain acyl-carnitines has been shown to decrease with age in the blood of mice [62].

Furthermore, studies have explored the role that L-carnitine presents as an anti-inflammatory, antioxidant and antiapoptotic agent. Specifically, scattered reports have shown that L-carnitine may suppress pro-inflammatory cytokines and control the inflammatory and immune response [24,65,66]. For example, the anti-inflammatory response of L-carnitine seems to be connected with its downregulated expression in RRD where inflammation development is present [25]. Moreover, experimental studies showed that L-carnitine could improve carbohydrate metabolism, reduce oxidative stress and free radical levels, and prevent subsequent cell death during ischemia [78], while other experimental models have indicated that L-carnitine, as well as its derivative, acetylcarnitine, have been shown to have neuroprotective, antioxidant, and antiapoptotic reactivity in the retinal cells of mouse models with high intraocular pressure [63]. Moreover, L-carnitine in the AH has been reported to play a role in maintaining AH homeostasis and osmosis [79]. An important issue in the studies that have been presented is the type of biospecimen, since the levels of free/acylcarnitines should be related to it. For example, in AMD patients, the higher level of free carnitine present in the AH leads to lower levels of its esterified form, such as acylcarnitine, than in the choroid-retina and plasma [14]. Furthermore, L-carnitine seems to play an important role in the osmoregulation of the tear fluid. Although tear fluid is known to be hyperosmolar in the DES, the levels of L-carnitine were found to be reduced. Decreased amounts of L-carnitine in the tear film may be related to increased concentrations of other tear solutes, to active transport across the cell membrane of ocular tissues and to lessen the retention of water in the aqueous layer, resulting in hyperevaporation, hyperosmolarity, reduced control of oxidative stress in the tear film, and increased apoptosis in the corneal surface. Therefore, L-carnitine might have a possible protective effect by preventing the damaging impact of a hypertonic tear film, while the reduced L-carnitine concentration in the tears of these patients could be consequential of the DES and may lend itself to the development of a future diagnostic test for the disease [27,80,81].

## 6. Conclusions and Future Directions

In conclusion, the literature data on the application of carnitine metabolites in ophthalmology seem promising, and carnitines may be proven useful tools in the treatment of ocular diseases. However, there are challenges to be faced in the field. Although the alterations of specific acylcarnitines and L-carnitine are evident, there seems to be no clear pattern yet. This could be easily explained since this is an evolving field with a small number of different studies regarding each ocular disease and biospecimen, and only few human samples. Studies with large numbers of patients should be designed and several factors, for example, the sex, the age or the medication, and other systemic diseases, should be taken into consideration. Specifically, systematic diseases, such as cardiovascular disease and the metabolic syndrome, could influence the levels of acylcarnitines in blood samples. Indeed, identifying acylcarnitines as systemic biomarkers remains a significant challenge because of the potential overlap or interaction between increased or decreased plasma levels of acylcarnitines in cases of the coexistence of the aforementioned systematic diseases and the ocular diseases. Moreover, the published studies were mainly with humans [11,12,13,14,15,16,17,18,19,20,21,22,23,24,25,26,27] and, according to our knowledge, only few animal studies have been reported in the literature [18,70,71,72]. The usual biospecimen was plasma/serum, but there are also a few studies where aqueous or vitreous humor or tears have been used. Specifically, aqueous humor was taken in three studies regarding AMD, POAG, and CRVO [14,22,23], vitreous humor was examined in two studies regarding DR and RRD [18,25], and tears were the specimen obtained in two studies regarding POAG and DES [24,27]. Although aqueous or vitreous humor are more difficult to be obtained than blood or tears, it would also be interesting if ocular tissues or fluid could be used in a greater extent in future studies. 

Due to its unique metabolome, eye specimen metabolic profiling shows promise as an application area [82]. Carnitines are a class of metabolites that could act as potential biomarkers and contribute to the early diagnosis and better understanding of the underlying mechanisms of the disease, and for this, future investigation is needed. 

## Figures and Tables

**Table 1:** General information about metabolic profiling studies performed for eye diseases focusing on carnitines.

Eye Disease	Study Participants	Carnitine Biomarker	Change	Bio- Specimen	Study Method/ Total Metabolites /Number (Type) of Carnitines	Analytical Technique	Ref.
AMD (wet)	AMD: 20 (27 eyes) Controls: 20	Palmitoylcarnitine (C16)	↓	Plasma	Untargeted	UHPLC-Q-TOF MS	[11]
AMD (wet)	NVAMD: 100* Controls: 192**	9-Hexadecenoylcarnitine (C16:1) Heptadecanoylcarnitine (C17) 11Z-octadecenylcarnitine (18:1) Palmitoylcarnitine (C16) Stearoylcarnitine (C18)	↑	Plasma	Untargeted	LC-MS/MS	[12]
AMD (wet)	Patients: 40 Controls: 40	L-carnitine (C0) Valerylcarnitine (C5)	↑	Plasma	Targeted/116/ 40 (C0 & 39 ACs)	FIA- MS /MS	[13]
AMD (wet)	Wet AMD patients: 26 (26 eyes) Controls: 20 (20 eyes)	L-carnitine (C0) Deoxycarnitine	↓ ↑	Aqueous humor	Untargeted	UHPLC-MS/MS	[14]
AMD (wet) vs. control NVAMD vs IAMD	IAMD: 91 NVAMD: 100 Controls: 195	Linoleylcarnitine (C18:2) Linolenylcarnitine (C18:3) Glutaconylcarnitine (C5:2) Heptadecanoylcarnitine (C17) 11Z-octadecenylcarnitine (C18:1) Stearoylcarnitine (C18)	↑ ↑ ↑ ↑ ↑ ↑	Plasma	Untargeted	LC-MS/MS	[15]
DR vs. DM PDR vs. NPDR	DR patients: 83 Controls: 90***	Dehydroxycarnitine L-carnitine (C0)	↑ ↑	Plasma	Untargeted	LC-MS	[16]
DR vs. NDR	NPDR: 123 PDR: 51 Controls: 143****	Propionylcarnitine (C3) Butyrylcarnitine (C4)	↑ ↑	Serum	Targeted/80/ 11 (C0 & ACs)	LC-MS, FIA-MS	[17]
		Dodecanoylcarnitine (C12) Tetradecenoylcarnitine(C14:1) Tetradecadienylcarnitine (C14:2) Hexadecanoylcarnitine (C16) Octadecenoylcarnitine(C18:1) Octadecadienylcarnitine (C18:2)	↓ ↓ ↓ ↓ ↓ ↓				
NPDR vs. NDR		L-carnitine (C0) Tetradecenoylcarnitine(C14:1) Hexadecanoylcarnitine (C16)	↓ ↓ ↓				
		Propionylcarnitine (C3) Butyrylcarnitine (C4)	↑ ↑				
PDR vs. NDR		Valerycarnitine (C5) Pimelycarnitine (C7 DC)	↓ ↓				
		Tetradecenoylcarnitine(C14:1) Hexadecanoylcarnitine (C16) Octadecanoylcarnitine (C18) Octadecenoylcarnitine(C18:1) Octadecadienylcarnitine(C18:2)	↓ ↓ ↓ ↓ ↓				
PDR and NPDR vs. NDR		Tetradecenoylcarnitine (C14:1) Hexadecanoylcarnitine (C16)	↓ ↓				
PDR vs. controls	PDR patients: 20 Controls: 31*****	Octanoylcarnitine (C8) Decanoylcarnitine (C10)	↑ ↑	Vitreous humor	Targeted/17/ 4 (C0 & 3ACs)	LC-MS/MS	[18]
ROP	ROP: 40 Controls: 41******	Malonylcarnitine (C3DC)	↑	Blood	Targeted/10/ 1 (ACs)	UPLC-MS	[19]
POAG	POAG: 72 Controls: 72	Palmitoylcarnitine (C16)	↑	Plasma	Untargeted	LC-MS	[20]
POAG	POAG: 36 Controls: 27*******	Octadecenoylcarnitine (C18:1) Propionylcarnitine (C3) Butyrylcarnitine (C4) Decanoylcarnitine (C10:1) Dodecanoylcarnitine (C12:1) Octadecadienylcarnitine (C18:2)	↓ ↑ ↑ ↑ ↑ ↑	Plasma	Targeted/151/ 14 (C0 & 13ACs)	FIA-MS	[21]
POAG	POAG: 26 Controls: 26*******	L-carnitine (C0) Acetylcarnitine (C2) Propionylcarnitine (C3) Butyrylcarnitine (C4)	↑ ↑ ↑ ↑	Aqueous humor	Targeted/54/ 4 (C0 & 3ACs)	FIA-MS/MS	[22]
POAG	POAG: 8 Controls: 16	L-carnitine (C0) Acetylcarnitine (C2) Propionylcarnitine (C3) Malonylcarnitine (C3-DC (C4-OH)) Butyrylcarnitine (C4) Butenoylcarnitine (C4:1) Valerylcarnitine (C5) Glutarylcarnine (C5-DC) Hydroxyhexanoylcarnitine (C6-OH) Hydroxypentanoylcarnitine (C5-OH) Malonylcarnitine (C3-DC-M) Pentenoylcarnitine (C5:1) Dodecanoylcarnitine (C12:1) Decatranoylcarnitine (C14:2-OH) Decanoylcarnitine (C10)	↑ ↑ ↑ ↑ ↑ - ↑ ↑ ↑ ↑ ↑ ↑ ↑ - ↓	Aqueous humor	Targeted/ 80/13(ACs)		[23]
POAG	POAG:16 Controls: 17	Acetylcarnitine (C2)	↓	Tears	Targeted/57/ 36 (C0 & 35As)	UPLC-MS/MS	[24]
RRD vs. RRD and PVR RRD or RRD and PVR vs. controls	RRD: 8 PVR: 9 Controls: 6	L-carnitine (C0)	↓	Vitreous humor	Targeted/31/ 1 (C0)	LC-Q-TOF-MS	[25]
CRVO vs. controls	CRVO: 15 (15 eyes) Controls: 20*******	L-carnitine (C0) Butyrylcarnitine (C4) Deoxycarnitine	↑ ↑ ↑	Aqueous humor	Untargeted	UHPLC-MS/MS HILIC	[26]
DES vs. controls	DES: 10 Controls: 10	Carnitine (C0) Acetylcarnitine (C2) Propionylcarnitine (C3)	↓ ↓ ↓	Tears	Targeted/3/ 3 (C0 & 2As)	HPLC-MS	[27]

AMD: Age-related macular disease; CRVO: Central retinal vein occlusion; DES: Dry eye syndrome; DM: Diabetes mellitus; DR: Diabetic retinopathy; NDR: Non-diabetic retinopathy; NPDR: Non-proliferative diabetic retinopathy; OIR: Oxygen-induced retinopathy; PDR: Proliferative diabetic retinopathy; POAG: Primary open-angle glaucoma; ROP: Retinopathy of prematurity; RRD: Rhegmatogenous retinal detachment. *92 grade 5 in both eyes, 8 grade 4 in the fellow eye; **AMD grade 1 in both eyes in which there are <10 drusen and no macular pigment changes; ***diabetic; ****NDR; *****non-diabetic, with epiretinal membrane or macular hole; ******non-ROP; *******with cataract. ACs: Acylcarnitines; UHPLC-Q-TOF: Ultra-high performance liquid chromatography-quadrupole-time-of-flight; MS: Mass spectrometry; LC: Liquid chromatography; MS/MS: Tandem mass spectrometry; FIA: Flow injection; UHPLC: Ultra-high performance liquid chromatography; UPLC: Ultra performance liquid chromatography; HILIC: Hydrophilic interaction liquid chromatography, HPLC: High performance liquid chromatography.

## Data Availability

Not applicable.
